# Adherence to guidelines-recommended diagnostic testing was associated with overall survival in patients with diffuse large B-cell lymphoma after rituximab-based treatment: an observational cohort study

**DOI:** 10.1007/s00432-022-04179-8

**Published:** 2022-08-17

**Authors:** Fei Yang, Ju Zhang, Anup Abraham, Jessie T. Yan, Richard D. Hammer, Matthew S. Prime

**Affiliations:** 1 Roche Information Solutions, Roche Diagnostics, Grenzacherstrasse 124, Building 71, CH-4070 Basel, Switzerland; 2 Roche Information Solutions, Roche Diagnostics, Santa Clara, CA USA; 3 Genesis Research, Hoboken, NJ USA; 4 Department of Pathology and Anatomical Sciences, University of Missouri, Columbia, MO USA

**Keywords:** DLBCL, Guideline adherence, Diagnostic testing, Overall survival

## Abstract

**Purpose:**

This study assessed the impact of adherence to guidelines-recommended diagnostic testing on treatment selection and overall survival (OS) in patients with diffuse large B-cell lymphoma (DLBCL) initiated on rituximab-based first line of treatment (1-LOT).

**Methods:**

This retrospective cohort study used a nationwide electronic health record-derived de-identified database, including diagnostic testing information on immunohistochemistry (IHC), fluorescence in situ hybridization (FISH) and karyotype analysis that were abstracted from pathology reports or clinical visit notes, where available. The study included patients above 18 years old who were diagnosed with DLBCL between January 2011 and December 2019 and initiated on rituximab-based 1-LOT. Patients were classified into ‘non-adherence,’ ‘partial-adherence’ and ‘complete-adherence’ groups according to the evidence/documentation of a confirmed known result for IHC and molecular profiling tests (FISH and karyotyping) on a selection of the markers prior to the initiation of 1-LOT. Logistic regression was used to evaluate associations of adherence to diagnostic testing with 1-LOT between R-CHOP and other rituximab-based regimens. Median OS after the start of rituximab-based 1-LOT was calculated using the Kaplan–Meier method. Multivariable-adjusted Cox proportional hazards regression was used to assess the risk of all-cause death after initiation of 1-LOT by the degrees of adherence to guidelines-recommended diagnostic testing.

**Results:**

In total, 3730 patients with DLBCL who initiated on rituximab-based 1-LOT were included. No association was found between adherence to guidelines-recommended diagnostic testing and treatment selection of 1-LOT for R-CHOP versus other rituximab-based regimens. Patients with a higher degree of adherence to guidelines-recommended diagnostic testing survived longer (median OS at 5.1, 6.9 and 7.1 years for ‘non-adherence,’ ‘partial-adherence’ and ‘complete-adherence’ groups, respectively [log-rank *p* < 0.001]) and had a decreased mortality risk (multivariable-adjusted hazard ratio with 95% confidence intervals at 0.83 [0.70–0.99] for ‘partial-adherence’ and 0.77 [0.64–0.91] for ‘complete-adherence’ groups, respectively).

**Conclusion:**

Patients’ adherence to guidelines-recommended diagnostic testing were associated with better survival benefit, reinforcing the need for adoption of diagnostic testing guidelines in routine clinical care.

**Supplementary Information:**

The online version contains supplementary material available at 10.1007/s00432-022-04179-8.

## Background

Diffuse large B-cell lymphoma (DLBCL) is the most common type of aggressive non-Hodgkin lymphoma (NHL) in adults, accounting for approximately 30–40% of NHL cases diagnosed annually with the incidence of 5.6 per 100,000 men and women per year in the United States (US) (SEER Cancer Stat Facts 2022). Since the introduction of rituximab two decades ago, the majority of DLBCL patients are curable with combination chemo-immunotherapy consisting of rituximab, cyclophosphamide, doxorubicin, vincristine and prednisone (R-CHOP) that has become the standard of care as the first line of treatment (1-LOT) (Coiffier et al. 2002; Seshadri et al. 2008; Zhang et al. 2018; NCCN Guidelines 2022). However, not everyone receives the same rituximab-based treatment and the prognosis and outcome can vary across DLBCL subtypes (Imhoff et al. 2006; Nowakowski and Czuczman 2015; Nowakowski et al. 2019; Susanibar-Adaniya and Barta 2021). To date, more than 30% DLBCL patients still die within five years (SEER Cancer Stat Facts 2022).

The disease is known to be clinically and molecularly heterogeneous and include different subtypes based on the cell of origin (COO) and other phenotypic and molecular/cytogenetic features (Nowakowski et al. 2019; Swerdlow et al. 2016; Alizadeh et al. 2000; Paepe and Wolf-Peeters 2007). For example, gene expression profiling (GEP) can identify two major and clinically distinct DLBCL subtypes based on COO, namely germinal center *B*-cell (GCB) subtype and non-GCB subtype (Hans 2004). DLBCL can also be classified into high-grade *B*-cell lymphomas with translocations involving *MYC* oncogene and *BCL2* and/or *BCL6* genes, previously called ‘double-/triple-hit’ lymphoma (HGBCL-DH/TH) (NCCN Guidelines 2022; Swerdlow et al. 2016).

Testing for DLBCL subtypes may help guide treatment selection enabling a more accurate prognosis for an improved outcome (Nowakowski et al. 2019; Rosenwald et al. 2002). National Comprehensive Cancer Network (NCCN) guidelines currently recommend immunohistochemistry (IHC) as a surrogate for GEP in clinical practice to help differentiate DLBCL between GCB and non-GCB subtypes (NCCN Guidelines 2022; Hans 2004). In addition, the guidelines recommend fluorescence in situ hybridization (FISH) or karyotype analysis for *MYC* gene rearrangement. Among those with a positive result for *MYC* gene rearrangement, the guidelines further suggest additional testing for the detection of *BCL2* and *BCL6* gene rearrangements.

However, it is unclear whether NCCN guidelines-recommended diagnostic testing for these biomarkers has been properly implemented in real-world clinical settings, and whether adherence to the testing impacts treatment decisions and patient outcomes. The objective of this study was to assess the associations between adherence to guidelines-recommended diagnostic testing and treatment selection as well as overall survival (OS) in DLBCL patients initiated on rituximab-based 1-LOT.

## Methods

### Data source, study design and population

**Fig. 1:**
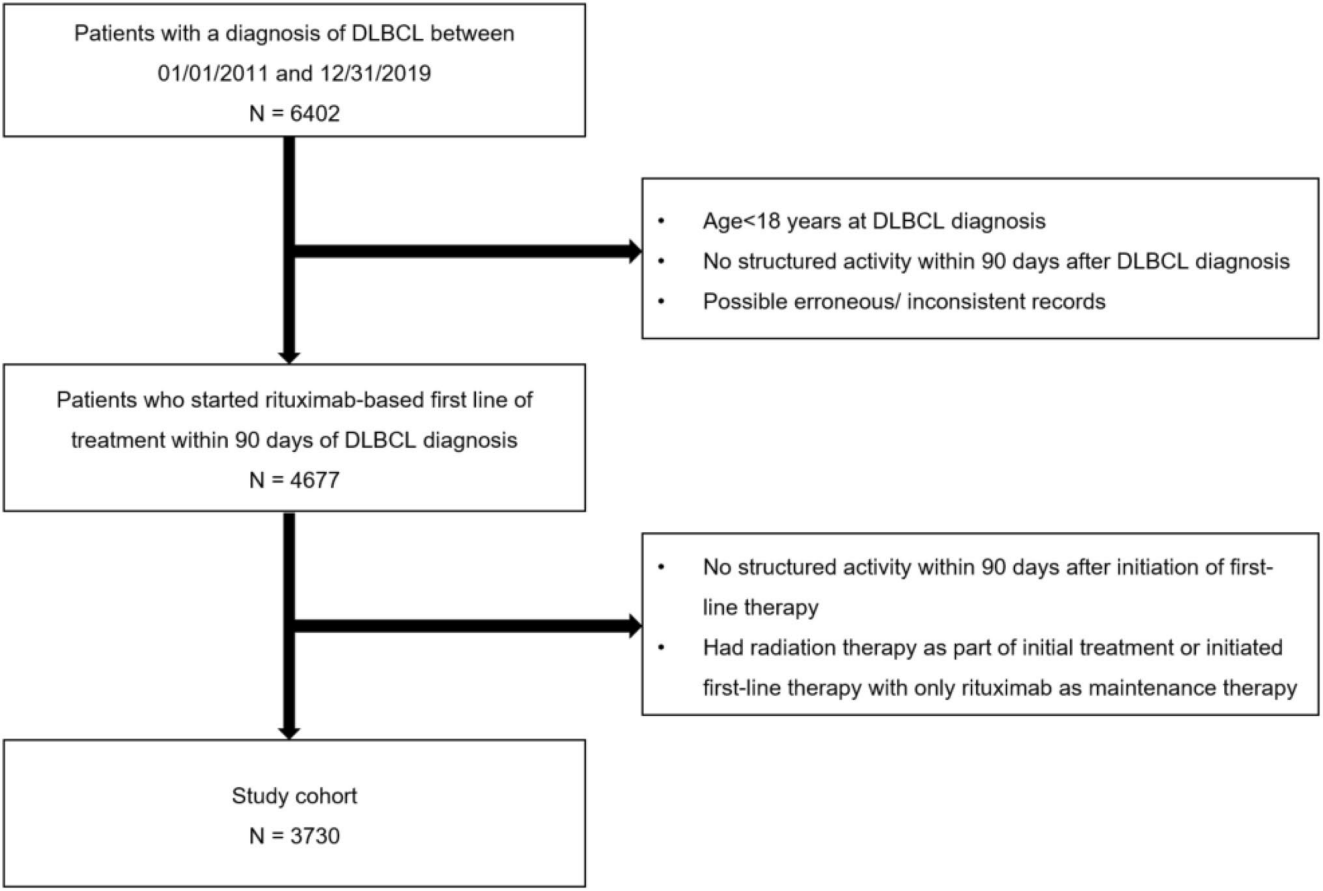
Flow Diagram of Study Population

This retrospective observational cohort study used nationwide longitudinal real-world data from the Flatiron Health electronic health record-derived de-identified database, comprising de-identified patient level structured and unstructured data, curated via technology-enabled abstraction (Ma et al. 2020; Birnbaum et al. 2020). During the study period, the de-identified data originated from approximately 280 cancer clinics (approximately 800 sites of care) in the USA (Zhang et al. 2021). The cohort included adult patients who had a DLBCL diagnosis documented between January 1, 2011, and December 31, 2019 (inclusive), with at least two documented clinical visits on or after January 1, 2011, and initiated rituximab-based 1-LOT within 90 days of DLBCL diagnosis. The line of therapy rules were oncologist-defined, rule-based line of therapy. Patients were excluded if they (1) had erroneous/inconsistent records (e.g., unknown sex, last structured activity before or on the day of DLBCL diagnosis, death date before structured activity date); (2) had no structured activity within 90 days of rituximab-based 1-LOT initiation; or (3) had radiation therapy as part of initial treatment for DLBCL or initiated 1-LOT with only rituximab as maintenance therapy. A total of 3730 DLBCL patients met the inclusion criteria for the analysis (Fig. 1).

The date of initiation of rituximab-based 1-LOT was defined as the *index date*. All patients were followed from the index date until death or loss to follow-up (censored at date of the last structured activity or abstracted oral therapy documented in the electronic health records, or end of the study on December 31, 2019).

### Demographic and clinical characteristics of patients

Patients’ demographic and clinical characteristics were measured on or around the index date, including age, gender, race/ethnicity, geographic region, type of clinical practice where the diagnosis was made, type of health insurance plan, year of DLBCL diagnosis, initial tumor group stage, whether DLBCL was transformed from a prior indolent lymphoid malignancy, status of lactate dehydrogenase (LDH) level within 30 days before and after the diagnosis, Eastern Cooperative Oncology Group (ECOG) performance status within 30 days before and after the diagnosis, whether there was extra-nodal site present at time of the diagnosis, and whether there was any history of other primary cancers.

### Adherence to guidelines-recommended diagnostic testing

Adherence to guidelines-recommended diagnostic testing was defined as a composite variable according to IHC and other molecular profiling tests including both FISH and karyotype for a selection of biomarkers prior to initiation of rituximab-based 1-LOT. The IHC panel included biomarkers (CD10, BCL6, MUM1, MYC and BCL2) used for identification of GCB and non-GCB subtypes as well as double-expressor lymphoma (DEL) (NCCN Guidelines 2022; Susanibar-Adaniya and Barta 2021; Hans 2004; Riedell and Smith 2018). The molecular profiling panel included *MYC*, *BCL2* and *BCL6* oncogenes for identification of HGBCL with *MYC* and *BCL2* and/or *BLC6* rearrangement (NCCN Guidelines 2022; Susanibar-Adaniya and Barta 2021; Swerdlow et al. 2016).

The magnitude of adherence to guidelines-recommended diagnostic testing was further classified into three categories, termed ‘non-adherence’ (i.e., no evidence/documentation of diagnostic testing on any biomarker from IHC or molecular profiling tests), ‘partial-adherence’ (i.e., evidence/documentation of confirmed known result for at least one biomarker from either IHC or molecular profiling tests), and ‘complete-adherence’ (i.e., evidence/documentation of confirmed known result for at least one biomarker from both IHC and molecular profiling tests).

### First-line treatment

Patients’ treatment selection was classified into the following two groups based on guidelines-recommended rituximab-based treatment regimens: R-CHOP versus (vs.) other rituximab-based regimens such as R-EPOCH (rituximab plus etoposide, prednisone, vincristine, cyclophosphamide and doxorubicin), R-CHOP-like regimens (i.e., R-CHOP with any additional biologic agents), R-Benda (rituximab-bendamustine) and rituximab single-agent monotherapy (NCCN Guidelines 2022).

### Overall survival

OS was calculated from the index date to the date of death of any cause or last follow-up. Dates of death in the Flatiron Health database were sourced from a composite mortality variable composed of electronic health record data linked to commercial mortality data and the Social Security Administration’s Death Master File (Zhang et al. 2021).

### Statistical analysis

Patients included in the study were described both in the full cohort overall and separately according to the degrees of adherence to guidelines-recommended diagnostic testing. Chi-square or Fisher’s exact tests were used for categorical variables, and ANOVA test was used for continuous variables.

Logistic regression was performed to determine the associations between adherence to guidelines-recommended diagnostic testing and treatment selection of 1-LOT for R-CHOP vs. other rituximab-based regimens. Regarding the survival analyses, Kaplan–Meier (unadjusted) survival curves were plotted and compared using the log-rank test. Then, a Cox proportional hazards regression model was used to estimate the hazard ratios (HRs) and 95% confidence intervals (CIs) for associations between the degrees of adherence to guidelines-recommended diagnostic testing and risk of all-cause death after initiation of rituximab-based 1-LOT. ‘Non-adherence’ group was treated as the reference in all analyses. Baseline patient demographic and clinical characteristics were included as covariates for adjustment for potential confounding. The impact of lack of documentation for certain covariates on the associations between the adherence to guidelines-recommended diagnostic testing and OS was also evaluated by evaluating the differences between including the unknown/not documented category in the Cox model and excluding them from the model. The proportional hazards assumption was checked using the Schoenfeld residuals method.

## Results

### Baseline characteristics, temporal trends of diagnostic testing and associations with selection of first-line treatment

Table 1 shows that 3730 DLBCL patients included in our study were predominantly above 60 years of age (72.6%), male (55.7%), non-Hispanic White (69.9%), commercially insured (38.6%), from the South region (40%), and being diagnosed at a community hospital (88.2%). The majority of patients had their DLBCL initially diagnosed at stage III or above (53.9%) and had no evidence of transforming from a prior indolent lymphoid malignancy (85.3%) or a present extranodal site (88.3%), or any other primary cancer history (87.6%). Among 1765 patients who had ECOG status measured during the baseline, 81.5% of those had ECOG less than 2.

**Table 1:** Baseline demographic and clinical characteristics of DLBCL patients, overall and stratified by adherence groups of diagnostic testing

	All DLBCL patients (*N* = 3730)		Non-adherence (*N* = 453)		Partial-adherence (*N* = 1483)		Complete-adherence (*N* = 1794)		*P* value*
Age at DLBCL diagnosis, years									< 0.01
Mean (SD)	66.7	13.1	68.6	13.3	67.1	13.4	65.9	12.7	
Median (IQR)	69	59–77	73	62–79	70	60–78	68.0	59–76	
Min, Max	19	85	20	85	20	85	19	85	
Sex (*N* %)									0.14
Male	2079	55.7%	246	54.3%	803	54.1%	1030	57.4%	
Female	1651	44.3%	207	45.7%	680	45.9%	764	42.6%	
Year of DLBCL diagnosis (*N,* %)									< 0.01
2011–2013	915	24.5%	167	36.9%	431	29.1%	317	17.7%	
2014–2016	1311	35.1%	155	34.2%	538	36.3%	618	34.4%	
2017–2019	1504	40.3%	131	28.9%	514	34.7%	859	47.9%	
Race/ethnicity (*N,* %)									0.45
White	2609	69.9%	325	71.7%	1036	69.9%	1248	69.6%	
Black or African American	223	6.0%	30	6.6%	82	5.5%	111	6.2%	
Asian	81	2.2%	5	1.1%	36	2.4%	40	2.2%	
Hispanic or latino	36	1.0%	6	1.3%	9	0.6%	21	1.2%	
Other race	450	12.1%	46	10.2%	181	12.2%	223	12.4%	
Unknown/not documented	331	8.9%	41	9.1%	139	9.4%	151	8.4%	
Geographic location** (*N*, %)									0.12
South	1492	40.0%	182	40.2%	570	38.4%	740	41.2%	
West	578	15.5%	61	13.5%	232	15.6%	285	15.9%	
Midwest	472	12.7%	55	12.1%	185	12.5%	232	12.9%	
Northeast	655	17.6%	76	16.8%	290	19.6%	289	16.1%	
Other territories	48	1.3%	6	1.3%	24	1.6%	18	1.0%	
Unknown/not documented	485	13.0%	73	16.1%	182	12.3%	230	12.8%	
Practice type (*N*, %)									0.39
Community	3290	88.2%	392	86.5%	1318	88.9%	1580	88.1%	
Academic	440	11.8%	61	13.5%	165	11.1%	214	11.9%	
Type of insurance plan (*N*,** %)**									< 0.01
Commercial	1439	38.6%	156	34.4%	533	35.9%	750	41.8%	
Medicare + Medicaid	766	20.5%	104	23.0%	303	20.4%	359	20.0%	
Other payers***	409	11.0%	39	8.6%	173	11.7%	197	11.0%	
Not insured	1116	29.9%	154	34.0%	474	32.0%	488	27.2%	
Tumor group stage (*N*, %)									0.69
Stage I & II	801	21.5%	91	20.1%	314	21.2%	396	22.1%	
Stage III & IV	2,012	53.9%	208	45.9%	784	52.9%	1,020	56.9%	
Unknown/not documented	917	24.6%	154	34.0%	385	26.0%	378	21.1%	
Transformed from a prior indolent lymphoid malignancy (*N*, %)									0.06
No (Unknown/not documented)	3181	85.3%	373	82.3%	1256	84.7%	1552	86.5%	
Yes	549	14.7%	80	17.7%	227	15.3%	242	13.5%	
Status of serum LDH level, ± 30 days (*N*, %)									< 0.01
Normal (≤ upper limit of the normal range)	1127	30.2%	109	24.1%	451	30.4%	568	31.7%	
Elevated (> upper limit of the normal range)	1250	33.5%	124	27.4%	455	30.7%	669	37.3%	
Unknown/not documented	1353	36.3%	220	48.6%	577	38.9%	557	31.0%	
ECOG status, ± 30 days (*N*, %)									< 0.01
< 2	1438	38.6%	113	24.9%	556	37.5%	769	42.9%	
≥ 2	327	8.8%	45	9.9%	121	8.2%	161	9.0%	
Unknown/not documented	1965	52.7%	295	65.1%	806	54.3%	864	48.2%	
Extranodal site present (*N*, %)									0.04
≤ 1	3294	88.3%	411	90.7%	1322	89.1%	1561	87.0%	
> 1	436	11.7%	42	9.3%	161	10.9%	233	13.0%	
Other primary cancer history (*N*, %)									< 0.01
No (Unknown/not documented)	3268	87.6%	377	83.2%	1292	87.1%	1599	89.1%	
Yes	462	12.4%	76	16.8%	191	12.9%	195	10.9%	

Percentages may not always add up to 100% due to rounding

*DLBCL* diffuse large B-cell lymphoma, *ECOG* Eastern Cooperative Oncology Group, *LDH* serum lactate dehydrogenase

*P values were derived from respective statistical test (ANOVA test for continuous variables and Chi-squared/Fisher’s exact test for categorical variables) among three adherence groups of diagnostic testing

** Geographic locations as follow

Midwest = IL, IN, MI, OH, WI, IA, KS, MN, MO, NE, ND, SD

Northeast = CT, ME, MA, NH, RI, VT, NJ, NY, PA

South = DE, DC, FL, GA, MD, NC, SC, VA, WV, AL, KY, MS, TN, AR, LA, OK, TX

West = AZ, MT, CO, ID, NV, NM, UT, WY, AK, CA, HI, OR, WA

Other territories = AS, FM, GU, MH, MP, PR, PW, VI

*** Other payers include type unknown, government/patient support program and self-pay, etc

**Fig. 2:**
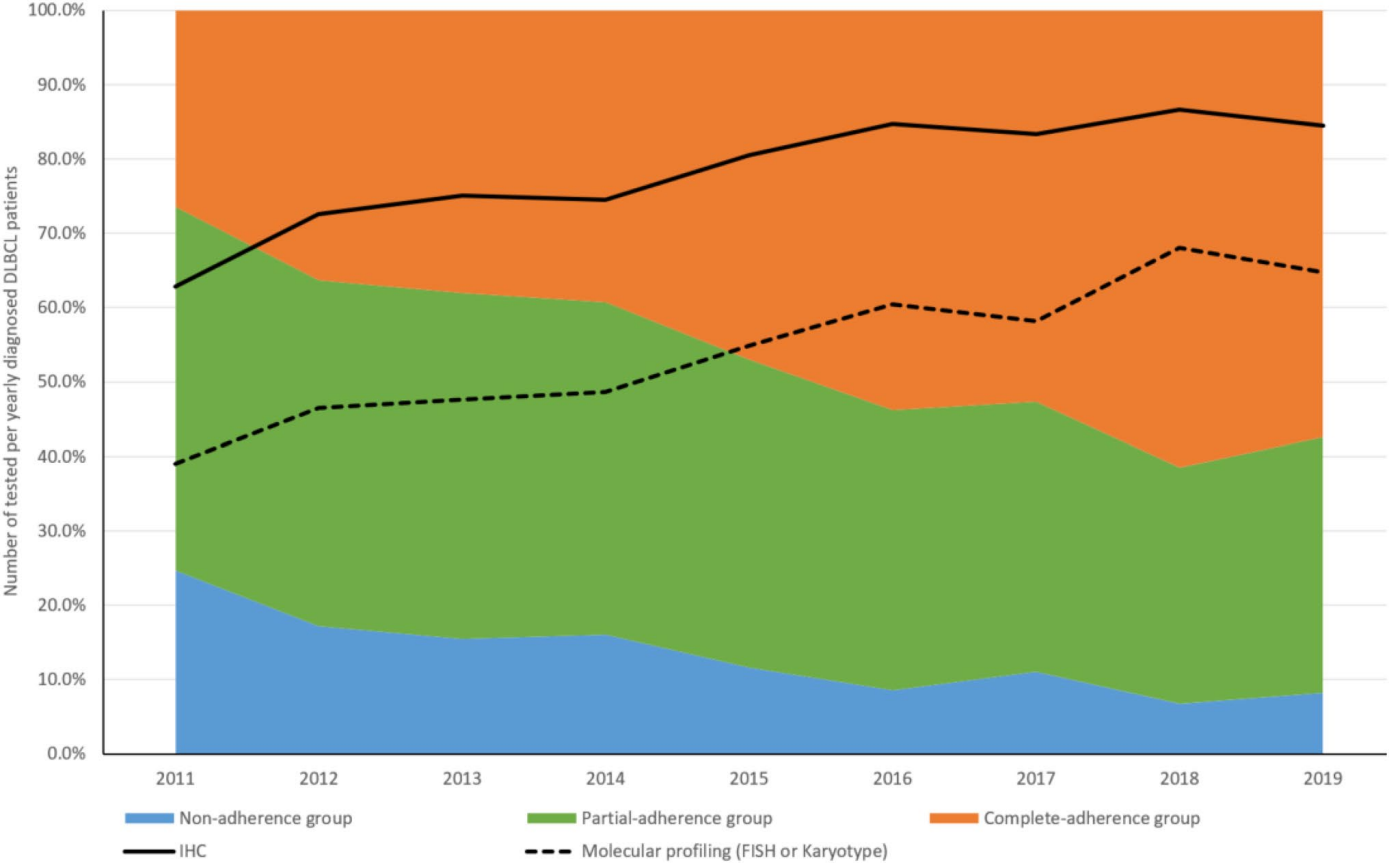
Trends of guidelines-recommended diagnostic testing prior to initiation of 1-LOT with rituximab-based treatment between 2011 and 2019. Abbreviations: *DLBCL* diffuse large B-cell lymphoma, *FISH*  fluorescence in situ hybridization, *IHC*  immunohistochemistry

Of all these DLBCL patients included in the study, guidelines-recommended diagnostic testing rates increased steadily between 2011 and 2019, from 62.8% to 84.5% for IHC and from 39.0% to 64.7% for molecular profiling tests (Fig. 2). In addition, the degrees of adherence to guidelines-recommended diagnostic testing also improved with more people classified as ‘partial-adherence’ and ‘complete-adherence’ during the years. Significant differences were also observed among the three adherence groups of diagnostic testing in baseline characteristics including age, type of insurance plan, serum LDH level, ECOG performance status, and whether there was extranodal site present and other primary cancer history at the time of DLBCL diagnosis (Table 1).

For initiation of 1-LOT, patients started with any rituximab-based treatment at a median of 23 days (IQR: 13–34 days) after the DLBCL diagnosis and initiated the R-CHOP regimen two times more than other rituximab-based regimens. However, no association was found between the degrees of adherence to guidelines-recommended diagnostic testing and selection of 1-LOT for R-CHOP vs. other rituximab-based regimens (Supplementary Material). In addition, there was no difference in the selection of 1-LOT between R-CHOP and other rituximab-based regimens when IHC or molecular profiling was considered separately.

### Overall survival analysis

**Fig. 3:**
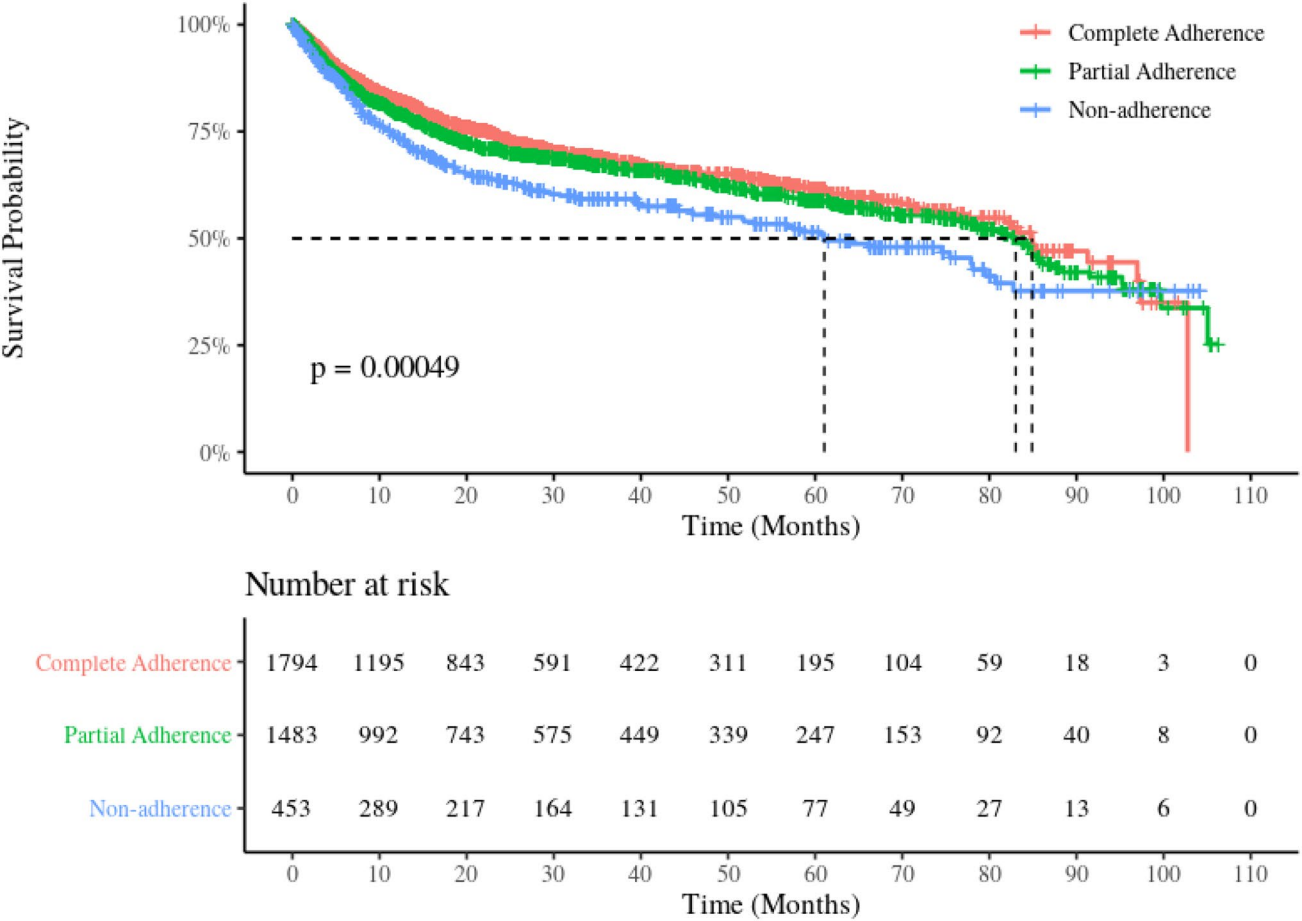
Unadjusted Kaplan–Meier curves of overall survival since initiation of rituximab-based first-line therapy by the degrees of adherence to guidelines-recommended diagnostic testing

There were 1155 patients who died with a median follow-up time of 18.9 months and the maximum length of follow-up was 107 months since the initiation of rituximab-based 1-LOT. When we looked at the impact of degrees of adherence to guidelines-recommended diagnostic testing on OS, as shown in the unadjusted Kaplan–Meier’s curves plotted in Fig. 3, the median OS was 5.1, 6.9, and 7.1 years for ‘non-adherence,’ ‘partial-adherence,’ and ‘complete-adherence’ groups, respectively (log-rank *p* < 0.001). Table 2 presents the results of the multivariable-adjusted Cox model and shows that compared to patients in the ‘non-adherence’ group, those with a ‘partial-adherence’ and ‘complete-adherence’ to guidelines-recommended diagnostic testing had lower risk of all-cause death (HR 0.83, 95% CI 0.70–0.99 for ‘partial-adherence’ group and HR 0.77, 95% CI 0.64–0.91 for ‘complete-adherence,’ respectively). The associations remained largely the same in the additional evaluation on potential impact of lack of documentation for certain covariates (Supplementary Material). Analyses of Schoenfeld’s residuals found no violation to the proportional hazards assumption (data not shown).

**Table 2:** Multivariable-adjusted HR and 95% CI estimates for all-cause death from initiation of first-line rituximab-based treatment by the degrees of adherence to guidelines-recommended diagnostic testing

Adherence groups of diagnostic testing	DLBCL patients		All-cause mortality*		
	Total (*N*)	Death (*N***)**	HR	95% CI	
Non-adherence	453	182	Reference		
Partial-adherence	1483	486	0.83	0.70	0.99
Complete-adherence	1794	487	0.77	0.64	0.91

*CI* confidence interval, *DLBCL*  diffuse large B-cell lymphoma, *HR*  hazard ratio

*Cox model was adjusted for all of the baseline patients’ demographic and clinical characteristics, including age (≤ 60 or > 60 years), gender, year of DLBCL diagnosis, race/ethnicity, geographic region, type of clinical practice where the diagnosis was made, type of health insurance plan, initial tumor group stage, whether DLBCL was transformed from a prior indolent lymphoid malignancy, status of lactate dehydrogenase level within 30 days before and after the diagnosis, Eastern Cooperative Oncology Group performance status within 30 days before and after the diagnosis, whether there was extra-nodal site present at time of the diagnosis, and whether there was any history of other primary cancers; Unknown/not documented was included as a separate category in the model

## Discussion

By analyzing a large national sample of adult patients diagnosed with DLBCL over a period of 9 years, our results show that there is strong evidence supporting adherence to NCCN biomarker testing guidelines: the majority (87.9%) of the patients received at least one NCCN guidelines-recommended diagnostic test of IHC or molecular profiling prior to their initiation of 1-LOT. Of these patients, more than half received both IHC and molecular profiling tests. About two-thirds of DLBCL patients initiated R-CHOP as their 1-LOT, but the treatment selection of 1-LOT between the R-CHOP and other rituximab-based regimens did not differ among the three adherence groups of diagnostic testing. However, compared to the ‘non-adherence’ patients who had no evidence of guidelines-recommended diagnostic test, ‘partial-adherence’ and ‘complete-adherence’ patients had a 17% and 23% reduction in the risk of all-cause death after initiation of 1-LOT, with a median OS of 22 and 23.9 months longer, respectively.

It should be noted that lymphomas consist of a remarkably diverse set of blood malignancies, which have traditionally been subdivided histologically. Thus, treatments have been developed primarily for each histological category of lymphoma irrespective of heterogeneous characteristics among the tumors in each category. NHL accounts for almost 90% of lymphoma cases, and DLBCL is the most common type of NHL with significant morphologic and cytogenetic heterogeneity (Paepe and Wolf-Peeters 2007; Lodhi et al. 2020). The NCCN guidelines currently suggest R-CHOP as the preferred 1-LOT for the majority of newly diagnosed DLBCL patients and recommend other rituximab-based regimens for those with certain risk factors (NCCN Guidelines 2022). Although a fairly large proportion of DLBCL patients respond well with survival rates similar to the general population in those who have remained disease-free for 2 years after frontline therapy of R-CHOP, approximately 30% of all DLBCL cases still experience limited clinical benefits (SEER Cancer Stat Facts 2022; Maurer et al. 2014; Jakobsen et al. 2017).

A tremendous need exists for improved diagnostic and treatment approaches for DLBCL patients. A more biomarker-driven precision medicine strategy could have a higher likelihood of success, because many studies have suggested that treatment outcomes may differ based on DLBCL subtypes (Lenz et al. 2008; Barrans et al. 2010). Ongoing efforts over the last three decades have tried to better understand the disease biology and have identified DLBCL subtypes at high-risk for failure to the standard of care. A landmark study in 2000 evaluated 96 normal and DLBCL lymphocytes using GEP and identified three unique genetic signatures based on COO (Alizadeh et al. 2000). It sparked the classification of DLBCL subtypes to predict prognosis and portended opportunities to optimize treatment selection and improve outcomes. In parallel to the COO classification system for DLBCL subtypes, molecular characteristics of DLBCL have also been found to have prognostic impacts, particularly with the advancement and recognition of FISH as the gold-standard technique for determining DNA rearrangements (Chapuy et al. 2018). However to date, GEP and some advanced techniques used in tumor analysis, such as next-generation sequencing (NGS) and high-resolution array comparative genomic hybridization, have not become part of routine clinical practice (Fukami and Miyado 2017; Jurczak et al. 2019).

It must be noted that diagnostic testing is important for precision cancer medicine. The advent of more conventional tests such as IHC and FISH is recommended in the NCCN guidelines and is fundamental toward precision medicine in routine clinical practice. Although our study could not shed light on the reasons for non-adherence to guidelines-recommended diagnostic testing nor did it reveal a difference in the selection of 1-LOT among the three adherence groups of diagnostic testing, the results suggested improved outcomes with a higher degree of adherence to the guidelines-recommended diagnostic testing. This highlights the importance of utilizing biomarker testing as an integral component of routine clinical practice. The recent pandemic of Coronavirus Disease 2019 (COVID-19) also presents the need of a more comprehensive and efficient cancer care decision-making process such as digital solutions of NAVIFY® Tumor Board and NAVIFY Guidelines that enable a better and flexible access to multidisciplinary and aggregated patient care data and up-to-date clinical guidance (Hammer and Prime 2020; Hammer et al. 2020). Streamlining and integrating these various methodologies in routine clinical practice will be an essential step toward precision medicine to improve DLBCL diagnosis and subsequent outcomes (Lodhi et al. 2020; Perry et al. 2012).

This study has some limitations. First, our study was biomarker-agnostic, meaning it did not focus on one specific biomarker but rather on a selection of biomarkers used by certain diagnostic testing methods for classification of some DLBCL subtypes. However, we tried to include the most important biomarkers used for subtype classification as recommended by the latest NCCN Guidelines. Second, biomarker diagnostic testing and OS in our study were assessed using real-world data, which may have been susceptible to unobserved biases that influenced the degree of adherence to diagnostic testing. Factors, such as concerns that treatment delays due to prospective biomarker testing, lack of biomarkers specific to DLBCL in the available lymphoma testing panels at the treating facility, and cost associated with the test might all play a role in whether patients adhere to guidelines-recommended diagnostic testing. Third, as we cannot uncover the reason of adherence to guidelines-recommended diagnostic testing, it is possible that the observed association of higher degrees of adherence with improved outcome could be a surrogate that represents a healthy and positive physician–patient care behavior that stimulates patient’s continuum of adherence to general cancer care. There could also be a potential for unmeasured bias on the selection of patients due to missing data or lack of documentation for certain data elements. Patients might have undergone guidelines-recommended diagnostic testing or received treatment outside of the Flatiron Health network. However, our evaluation on the potential effect of missing data was minimal on the associations between adherence to guidelines-recommended diagnostic testing and OS after initiation of rituximab-based 1-LOT.

## Conclusions

This study assessed adherence to guidelines-recommended diagnostic testing and its impact on treatment selection and clinical outcomes among DLBCL patients in the real-world setting in the USA. The study shows that guidelines-recommended diagnostic testing increased over time but continued improvement would still be needed, especially for molecular profiling test. Although better adherence to guidelines-recommended diagnostic testing appeared not to influence the selection of rituximab-based 1-LOT, it was associated with an improved OS after the treatment initiation.

## Supplementary Information

Below is the link to the electronic supplementary material.

Supplementary file1 (DOCX 22 KB)

## Data Availability

The data that support the findings of this study have been originated by Flatiron Health, Inc. These de-identified data may be made available upon request and are subject to a license agreement with Flatiron Health; interested researchers should contact < DataAccess@flatiron.com > to determine licensing terms.

## Abbreviations

1-LOT: First line of treatment
CI: Confidence interval
COVID-19: Coronavirus disease 2019
COO: Cell of origin
DEL: Double-expressor lymphoma
DH/TH: Double-/triple-hit
DLBCL: Diffuse large B-cell lymphoma
ECOG: Eastern cooperative oncology group
FISH: Fluorescence in situ hybridization
GCB: Germinal center B-cell
GEP: Gene expression profiling
HGBCL: High-grade B-cell lymphomas
HR: Hazard ratio
IHC: Immunohistochemistry
LDH: Lactate dehydrogenase
NCCN: National comprehensive cancer network
NHL: Non-hodgkin lymphoma
OS: Overall survival
R-CHOP: Rituximab, cyclophosphamide, doxorubicin, vincristine, and prednisone
USA: United States of America
