# Supplementary material for: Adherence to guidelines-recommended diagnostic testing was associated with overall survival in patients with diffuse large B-cell lymphoma after rituximab-based treatment: an observational cohort study
Source: J Cancer Res Clin Oncol. 2022 Aug 17;149(7):3691–700. doi: 10.1007/s00432-022-04179-8 (PMC9381398; doi:10.1007/s00432-022-04179-8)
Supplement: Supplementary file 1 — Supplementary file1 (DOCX 22 KB) [file 432_2022_4179_MOESM1_ESM.docx]

# Supplementary Material

**Supplementary Table S1: Associations between adherence to guidelines-recommended diagnostic testing and treatment selection of first-line rituximab-based treatment for R-CHOP versus other rituximab-based regimens**

|  | **First-line rituximab-based treatment** | | | | | **Univariate analysis**** | | |
| --- | --- | --- | --- | --- | --- | --- | --- | --- |
|  | **R-CHOP**  **(N=2486)** | | **Other rituximab-based regimens**  **(N=1244)** | | **P-value*** |  |  |  |
|  | **N** | **%** | **N** | **%** |  | **OR** | **95% CI** | |
| **Adherence groups of diagnostic testing** | | | | | 0.187 |  | | |
| Non-adherence | 292 | 11.75% | 161 | 12.94% |  | Reference | | |
| Partial-adherence | 1013 | 40.75% | 470 | 37.78% |  | 1.19 | 0.95 | 1.48 |
| Complete-adherence | 1181 | 47.51% | 613 | 49.28% |  | 1.06 | 0.85 | 1.32 |
| **IHC** | | | | | 0.417 |  | | |
| No evidence of testing | 490 | 19.71% | 260 | 20.90% |  | Reference | | |
| Tested | 1996 | 80.29% | 984 | 79.10% |  | 1.08 | 0.91 | 1.27 |
| **Molecular profiling (FISH and karyotyping)** | | | | | 0.323 |  | | |
| No evidence of testing | 1107 | 44.53% | 532 | 42.77% |  | Reference | | |
| Tested | 1379 | 55.47% | 712 | 57.23% |  | 0.93 | 0.81 | 1.07 |
| Abbreviations:  CI = confidence interval; FISH = fluorescence in situ hybridization; IHC = immunohistochemistry; OR = odds ratio.  Notes:  * P-values were derived from chi-squared test.  ** Logistic regression with the treatment selection of first-line rituximab-based treatment as the dependent variable. | | | | | | | | |

**Supplementary Table S2: Results from different Cox models for the multivariable-adjusted HR and 95% CI estimates for all-cause death from initiation of first-line rituximab-based treatment by the degrees of adherence to guidelines-recommended diagnostic testing**

|  | **Additional Cox model 1***  **(N=903)** | | | | **Additional Cox model 2***  **(N=3730)** | | | | **Additional Cox model 3***  **(N=2265)** | | | |
| --- | --- | --- | --- | --- | --- | --- | --- | --- | --- | --- | --- | --- |
|  | **n** | **HR** | **95% CI** | | **n** | **HR** | **95% CI** | | **n** | **HR** | **95% CI** | |
| **Adherence groups of diagnostic testing** | | | | | | | | | | | | |
| Non-adherence | 63 | Reference | | | 453 | Reference | | | 243 | Reference | | |
| Partial-adherence | 327 | 1.28 | 0.78 | 2.09 | 1483 | 0.79 | 0.67 | 0.94 | 889 | 0.85 | 0.67 | 1.08 |
| Complete-adherence | 513 | 0.93 | 0.57 | 1.52 | 1794 | 0.73 | 0.61 | 0.87 | 1133 | 0.70 | 0.55 | 0.90 |
| **Age group at DLBCL diagnosis, years** | | | | | | | | | | | | |
| ≤60 yr | 226 | Reference | | | 1021 | Reference | | | 644 | Reference | | |
| >60 yr | 677 | 1.99 | 1.34 | 2.95 | 2709 | 2.20 | 1.86 | 2.59 | 1621 | 2.22 | 1.80 | 2.75 |
| **Sex** | | | | | | | | | | | | |
| Male | 513 | Reference | | | 2079 | Reference | | | 1252 | Reference | | |
| Female | 390 | 0.70 | 0.54 | 0.91 | 1651 | 0.86 | 0.76 | 0.97 | 1013 | 0.86 | 0.74 | 1.01 |
| **Year of DLBCL diagnosis** | | | | | | | | | | | | |
| 2011-2013 | 140 | Reference | | | 915 | Reference | | | 586 | Reference | | |
| 2014-2016 | 340 | 1.01 | 0.71 | 1.44 | 1311 | 1.22 | 1.06 | 1.41 | 851 | 1.27 | 1.05 | 1.53 |
| 2017-2019 | 423 | 1.17 | 0.79 | 1.73 | 1504 | 1.15 | 0.97 | 1.36 | 828 | 1.25 | 1.00 | 1.58 |
| **Race/ethnicity** | | | | | | | | | | | | |
| White | 723 | Reference | | | 2609 | Reference | | | 1728 | Reference | | |
| Black or African American | 41 | 0.33 | 0.13 | 0.81 | 223 | 0.82 | 0.63 | 1.08 | 142 | 0.77 | 0.54 | 1.11 |
| Asian | 23 | 0.85 | 0.34 | 2.15 | 81 | 0.98 | 0.63 | 1.53 | 57 | 0.82 | 0.47 | 1.45 |
| Hispanic or Latino | 3 | 2.09 | 0.28 | 15.52 | 36 | 0.94 | 0.48 | 1.82 | 21 | 0.53 | 0.17 | 1.65 |
| Other Race | 113 | 0.61 | 0.38 | 0.99 | 450 | 0.70 | 0.57 | 0.87 | 317 | 0.68 | 0.52 | 0.88 |
| Unknown/not documented | N/A | | | | 331 | 1.49 | 1.22 | 1.81 | N/A | | | |
| **Geographic location**** | | | | | | | | | | | | |
| South | 455 | Reference | | | 1492 | Reference | | | 1045 | Reference | | |
| West | 124 | 0.74 | 0.49 | 1.11 | 578 | 1.09 | 0.92 | 1.31 | 380 | 0.98 | 0.78 | 1.23 |
| Midwest | 164 | 0.54 | 0.37 | 0.78 | 472 | 0.91 | 0.75 | 1.09 | 360 | 0.78 | 0.62 | 0.99 |
| Northeast | 155 | 0.77 | 0.53 | 1.11 | 655 | 0.95 | 0.80 | 1.11 | 450 | 1.00 | 0.82 | 1.22 |
| Other territories | 5 | 0.00 | 0.00 | Inf | 48 | 0.51 | 0.22 | 1.14 | 30 | 0.46 | 0.15 | 1.44 |
| Unknown/not documented | N/A | | | | 485 | 1.63 | 1.09 | 2.46 | N/A | | | |
| **Practice type** | | | | | | | | | | | | |
| Community | N/A | | | | 3290 | Reference | | | N/A | | | |
| Academic |  |  |  |  | 440 | 0.41 | 0.27 | 0.64 |  |  |  |  |
| **Type of insurance plan** | | | | | | | | | | | | |
| Commercial | 375 | Reference | | | 1439 | Reference | | | 875 | Reference | | |
| Medicare + Medicaid | 216 | 0.84 | 0.61 | 1.16 | 766 | 0.98 | 0.84 | 1.14 | 473 | 0.87 | 0.71 | 1.07 |
| Other payers*** | 76 | 0.85 | 0.50 | 1.44 | 409 | 0.86 | 0.69 | 1.07 | 225 | 0.80 | 0.59 | 1.09 |
| Not insured | 236 | 0.91 | 0.65 | 1.26 | 1116 | 1.02 | 0.88 | 1.18 | 692 | 0.99 | 0.82 | 1.20 |
| **Tumor group stage** | | | | | | | | | | | | |
| Stage I & II | 243 | Reference | | | 801 | Reference | | | 654 | Reference | | |
| Stage III & IV | 660 | 1.63 | 1.13 | 2.34 | 2012 | 1.58 | 1.33 | 1.88 | 1611 | 1.59 | 1.31 | 1.93 |
| Unknown/not documented | N/A | | | | 917 | 2.13 | 1.76 | 2.58 | N/A | | | |
| **Transformed from a prior indolent lymphoid malignancy** | | | | | | | | | | | | |
| No (Unknown/not documented) | 784 | Reference | | | 3181 | Reference | | | 2011 | Reference | | |
| Yes | 119 | 1.18 | 0.84 | 1.66 | 549 | 1.08 | 0.92 | 1.26 | 254 | 0.99 | 0.79 | 1.25 |
| **Status of serum LDH level, +/- 30 days** | | | | | | | | | | | | |
| Normal (≤ upper limit of the normal range) | 444 | Reference | | | N/A | | | | N/A | | | |
| Elevated (> upper limit of the normal range) | 459 | 1.96 | 1.49 | 2.58 |  |  |  |  |  |  |  |  |
| Unknown/not documented | N/A | | | |  |  |  |  |  |  |  |  |
| **ECOG status, +/- 30 days** | | | | | | | | | | | | |
| <2 | 762 | Reference | | | N/A | | | | N/A | | | |
| ≥2 | 141 | 1.98 | 1.46 | 2.68 |  |  |  |  |  |  |  |  |
| Unknown/not documented | N/A | | | |  |  |  |  |  |  |  |  |
| **Extranodal site present** | | | | | | | | | | | | |
| ≤1 | 803 | Reference | | | 3294 | Reference | | | 1989 | Reference | | |
| >1 | 100 | 0.99 | 0.68 | 1.46 | 436 | 1.26 | 1.06 | 1.49 | 276 | 1.19 | 0.95 | 1.48 |
| **Other primary cancer history** | | | | | | | | | | | | |
| No (Unknown/not documented) | 807 | Reference | | | 3268 | Reference | | | 2041 | Reference | | |
| Yes | 96 | 1.15 | 0.78 | 1.68 | 462 | 1.14 | 0.97 | 1.35 | 224 | 1.03 | 0.81 | 1.31 |
| Abbreviations:  CI = confidence interval; DLBCL = diffuse large B-cell lymphoma; ECOG = Eastern Cooperative Oncology Group; HR = hazard ratio; LDH = serum lactate dehydrogenase; N/A = not applicable (categories/variables not included in the model).  Notes:  * Different combinations of covariates were adjusted in the Cox models to evaluate potential impact of lack of documentation for certain covariates on the associations between the adherence to guidelines-recommended diagnostic testing and OS.  ** Geographic locations as follow:  Midwest = IL, IN, MI, OH, WI, IA, KS, MN, MO, NE, ND, SD Northeast = CT, ME, MA, NH, RI, VT, NJ, NY, PA South = DE, DC, FL, GA, MD, NC, SC, VA, WV, AL, KY, MS, TN, AR, LA, OK, TX West = AZ, MT, CO, ID, NV, NM, UT, WY, AK, CA, HI, OR, WA Other territories = AS, FM, GU, MH, MP, PR, PW, VI  *** Other payers include type unknown, government/patient support program and self pay, etc. | | | | | | | | | | | | |
